# Tamoxifen-Activated CreERT Impairs Retinal Angiogenesis Independently of Gene Deletion

**DOI:** 10.1161/CIRCRESAHA.120.317025

**Published:** 2020-07-08

**Authors:** James T. Brash, Rebecca L. Bolton, Victoria S. Rashbrook, Laura Denti, Yoshiaki Kubota, Christiana Ruhrberg

**Affiliations:** 1 From the UCL Institute of Ophthalmology, University College London, United Kingdom (J.T.B., R.L.B., V.S.R., L.D., C.R.); 2 Department of Anatomy, Keio University School of Medicine, Shinjuku-ku, Tokyo, Japan (Y.K.).

**Keywords:** Cre recombinase, endothelial cell, mutation, retina, tamoxifen

**Meet the First Author, see p 706**

The perinatal mouse retina is the current model of choice to define the mechanisms of sprouting angiogenesis.^1^ After birth, blood vessels grow from the optic nerve head towards the retinal periphery while branching laterally to establish a planar network that is exquisitely suited for quantifying vascular extension and branching.^1^ Cre recombinase–mediated ablation of loxP-flanked (floxed) genes is widely used to uncover angiogenesis genes in the mouse retina, with temporal control available through the Cre recombinase estrogen fusion protein with ligand binding mutation (CreER), whose activity is tamoxifen-dependent.^2,3^ The correct interpretation of experiments employing CreER-mediated gene ablation requires controls to exclude off-target effects, such as transgene insertion into essential genomic loci or toxic effects of activating CreER. Despite reports of impaired cell proliferation and DNA damage in several cell types after CreER activation,^4^ published work has not addressed whether CreER toxicity affects the endothelial cells that line all blood vessels and drive angiogenesis.

We have compared retinal angiogenesis in littermate perinatal day 7 pups expressing or lacking the endothelial cell–selective *Cdh5-CreER*^*T2*^ transgene with the Mouse Genome Informatics identifier 5705396.^5^ Mice were injected at perinatal day 2 and 4 with peanut oil or peanut oil containing tamoxifen doses representative of those commonly reported.^3^ Neither CreER^T2^ nor tamoxifen alone impaired vascular network extension or branch density (Figure [A] and [B]). In contrast, and despite the absence of floxed target genes, both vascular parameters were significantly reduced in the retinas of tamoxifen-injected pups expressing CreER^T2^ compared with tamoxifen-injected pups lacking CreER^T2^ (Figure [A] and [B]; similar defects were seen with the tamoxifen metabolite 4-hydroxytamoxifen; data not shown). Linear regression analysis revealed a significant, negative relationship of both vascular parameters with tamoxifen dose in *Cdh5-CreER*^*T2*^ mice (Figure [A] and [B]; extension: regression coefficient, −0.00095, *R*^2=^0.46, *P*=1.69×10^−5^; branch density: regression coefficient, −0.0026, *R*^2=^0.54, *P*=1.69×10^−6^). Vascular network extension and branch density were also significantly reduced in the retinas of tamoxifen-injected pups carrying the tamoxifen-inducible, endothelial cell–selective *Pdgfb-iCreER*^*T2*^ transgene (Mouse Genome Informatics identifier 3793852^5^; Figure [C] and [D]). The bodyweight of CreER^T2^ mice was not reduced compared to CreER^T2^-deficient littermates, excluding that a general developmental delay had impeded angiogenesis (data not shown). We conclude that tamoxifen-activated CreER^T2^ compromises retinal angiogenesis. In contrast, *Tie2-Cre*, which is constitutively expressed in endothelial cells (Mouse Genome Informatics identifier 2450311),^5^ did not impair retinal angiogenesis (Figure [C] and [D]), although this does not exclude toxicity of constitutive Cre activity in other circumstances.

**Figure.**
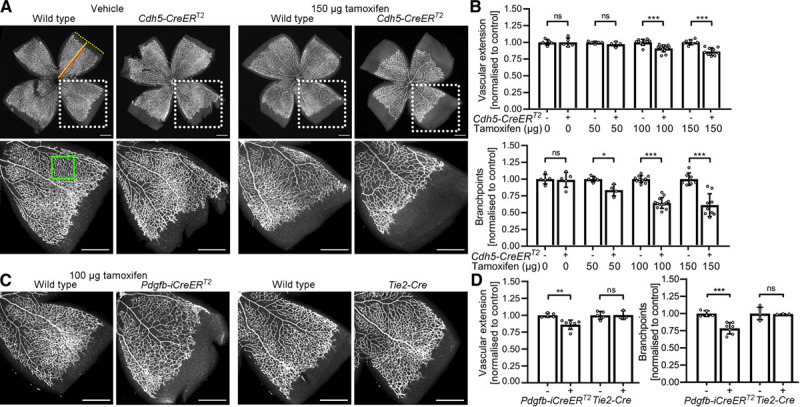
**Endothelial Cre recombinase estrogen fusion protein with ligand binding mutation (CreER)^T2^ activity impairs retinal angiogenesis**. Flat mounted perinatal day (P) 7 retinas were stained with the vascular endothelial marker IB4 (isolectin B4) and fluorescent streptavidin.^3^ Scale bars: 500 µm. **A** and **B**, *Cdh5-CreER*^*T2*^–expressing and wildtype littermates were injected at P2 and P4 with 25 µL peanut oil containing 0, 50, 100, or 150 µg tamoxifen. **A**, Micrographs and (**B**) quantification of vascular extension and branch density. Dotted boxes indicate areas shown at higher magnification. Red and yellow lines indicate vascular extension and retinal radius. The green box indicates a representative area analyzed for vascular branch density. **C** and **D**, *Pdgfb-iCreER*^*T2*^–expressing and wildtype littermates were injected at P2 and P4 with 25 µL peanut oil containing 100 µg tamoxifen. *Tie2-Cre* litters were not injected. **C**, Micrographs and (**D**) quantification of vascular extension and branch density. Data are presented as mean±SD fold change relative to littermate controls; each data point represents the average of several retinal leaflets. *Cdh5-CreER*^*T2*^ experiments: controls n=5 (0 µg), n=5 (50 µg), n=10 (100 µg), n=7 (150 µg); *CreER*^*T2*^ n=5 (0 µg), n=4 (50 µg), n=13 (100 µg), n=9 (150 µg); *Pdgfb-iCreER*^*T2*^ experiments: controls n=5, *CreER*^*T2*^ n=7; *Tie2-Cre* experiments: controls n=5, *Tie2-Cre* n=3. Two-way ANOVA with Holm-Sidak multiple comparison test, nonsignificant (ns), *P*>0.05; *P<0.05; **P<0.01, ***P<0.001.

Our findings imply that CreER toxicity has likely confounded the interpretation of some prior retinal angiogenesis studies, including our own, because they have compared tamoxifen-injected CreER-expressing pups to treatment-naive or tamoxifen-injected CreER-negative littermates. As CreER-mediated gene targeting remains a key method for angiogenesis research, we propose that future retinal studies should include tamoxifen-injected CreER control mice lacking floxed genes. Moreover, potential CreER toxicity should be considered when studying angiogenesis in other organs.

## Sources of Funding

This work was supported by the British Heart Foundation (FS/16/61/32740, FS/18/65/34186, PG/19/37/3439) and Wellcome (205099/Z/16/Z). This study used mice on a C57/Bl6J background and was approved by the local Animal Welfare Ethical Review Body and the UK Home Office. The authors declare that data will be made available upon reasonable request.

## Disclosures

None.
